# Quantification of masking risk in screening mammography with volumetric breast density maps

**DOI:** 10.1007/s10549-017-4137-4

**Published:** 2017-02-04

**Authors:** Katharina Holland, Carla H. van Gils, Ritse M. Mann, Nico Karssemeijer

**Affiliations:** 1 Department of Radiology and Nuclear Medicine, Radboud University Medical Center, PO Box 9101, 6500 HB Nijmegen, The Netherlands; 2 Julius Center for Health Sciences and Primary Care, University Medical Center Utrecht, PO Box 85500, 3508 GA Utrecht, The Netherlands

**Keywords:** Breast cancer screening, Volumetric breast density, Masking, Risk stratification, Supplemental screening

## Abstract

**Purpose:**

Fibroglandular tissue may mask breast cancers, thereby reducing the sensitivity of mammography. Here, we investigate methods for identification of women at high risk of a masked tumor, who could benefit from additional imaging.

**Methods:**

The last negative screening mammograms of 111 women with interval cancer (IC) within 12 months after the examination and 1110 selected normal screening exams from women without cancer were used. From the mammograms, volumetric breast density maps were computed, which provide the dense tissue thickness for each pixel location. With these maps, three measurements were derived: (1) percent dense volume (PDV), (2) percent area where dense tissue thickness exceeds 1 cm (PDA), and (3) dense tissue masking model (DTMM). Breast density was scored by a breast radiologist using BI-RADS. Women with heterogeneously and extremely dense breasts were considered at high masking risk. For each masking measure, mammograms were divided into a high- and low-risk category such that the same proportion of the controls is at high masking risk as with BI-RADS.

**Results:**

Of the women with IC, 66.1, 71.9, 69.2, and 63.0% were categorized to be at high masking risk with PDV, PDA, DTMM, and BI-RADS, respectively, against 38.5% of the controls. The proportion of IC at high masking risk is statistically significantly different between BI-RADS and PDA (*p*-value 0.022). Differences between BI-RADS and PDV, or BI-RADS and DTMM, are not statistically significant.

**Conclusion:**

Measures based on density maps, and in particular PDA, are promising tools to identify women at high risk for a masked cancer.

## Introduction

Thanks to screening programs, breast cancers are often detected at an early stage. Nevertheless, not all breast cancers in breast cancer screening participants are actually detected by screening. Approximately 16–33% of the breast cancer cases are the so-called interval cancers, which means that they are diagnosed in between two screening rounds [1, 2], even though the introduction of digital mammography may have led to an increase in sensitivity [3, 4]. In general, interval cancers are detected at a later stage and have a worse prognosis [5–7]. Fibroglandular tissue may mask cancers, and therefore sensitivity of mammography decreases with an increase in breast density. It has been shown that there is a relationship between breast density and screening program sensitivity [8–13]. In addition, compared to women in the lowest density category, women with dense breasts also have a higher breast cancer risk [14–16], which amplifies the negative effect of masking.

To detect more cancers at an early stage, personalized screening programs have been proposed [17, 18]. Adjusted to the personal needs of individual women, screening could be offered with different time intervals or with other modalities than mammography, such as ultrasound or MRI. Tomosynthesis might be an option as well, although the effect is limited for extremely dense breasts [19]. In this discussion, the reduced sensitivity of mammography due to the masking effect of density plays an important role. In recent years, many states in the United States passed breast density notifications laws. Radiologists are obliged to inform women about their breast density and the affiliated risks. In some states, additional imaging is reimbursed for women with dense breasts.

For the measurement of breast density, several methods are available. In clinical practice, the 4-point ACR BI-RADS scale is commonly used [20, 21]. To make this estimate less subjective, algorithms have been developed to estimate the breast density by computing the percentage dense area projected on the mammogram or by computing the percentage of fibroglandular tissue volume within the breast. An overview of different algorithms is presented by He [22].

Although breast density relates to masking, the relation between the risk of masking and density is likely to be more complex than a simple dependence on the amount of fibroglandular tissue. Also, the distribution of dense tissue may play a role. This is reflected in the new BI-RADS definition that no longer considers the total amount of fibroglandular tissue within the breast, but rather the densest area [21]. How the risk of masking should be quantified is an open question. The aim of this study is to compare three different quantitative masking measurements and the visual BI-RADS density assessment of a radiologist, in their ability to predict the risk of an interval cancer.

## Materials and methods

### Data

Digital mammograms from the Dutch breast cancer screening program were analyzed. The mammograms were acquired on Lorad Selenia systems (Hologic, Bedford, USA). Women aged 50–75 years are invited biennially to participate in the screening program. Details about the screening program and the dataset can be found elsewhere [23–25]. Written informed consent was not required for this study. Women automatically consent to the use of their anonymized data for scientific purposes by participating in screening. Data of participants who objected to the use of their data were removed.

The research archive used contains unprocessed mammograms of one screening unit. In the period 2003–2012, more than 130,000 exams of more than 55,000 women were acquired. Mediolateral oblique (MLO) images were always taken, while craniocaudal (CC) images were taken in the first screening round and in 60% of subsequent rounds. Through linkage with the Netherlands cancer registry and the screening organization, 1210 breast cancers were identified, of which 836 were screen-detected cancers. The remaining 374 breast cancers were diagnosed outside the screening program. Of these interval cancers, 275 were diagnosed within 24 months (screening interval), of which 113 cancers within 12 months after the examination. The last available screening examination before cancer diagnosis is used in this study. Women with breast implants were excluded from the study as the density maps cannot be correctly computed for mammograms with implants.

In this study, a selection of the interval cancers, the cancers that were diagnosed within 12 months after the examination, is used (*N* = 111, two women were excluded because of breast implants). The reason is that we want to focus on false negative exams due to masking. Interval cancers may also be due to other factors. In particular, fast growing cancers may not be detectable at the time of screening because they still are too small or not yet invasive. We assume that by excluding interval cancers detected more than 12 months after screening, a larger proportion of the interval cancers are due to masking. This idea is supported by Weber et al. [26] who found that a larger proportion of the interval cancers found in the second year after the screening examination show no signs of abnormality in the screening mammogram compared to the interval cancers found in the first year.

For each patient with an interval cancer, 10 participants were chosen as controls. The control participants needed to have had a mammographic examination in the same month in which the last screening examination of the interval cancer patient was performed. To be eligible as control, the women should not have been recalled on the basis of this mammographic examination and they should not have been diagnosed with breast cancer within 2 years after this examination. Women with breast implants were not eligible as control. Controls without a density map, due to failure of the computation, were replaced.

### Methods

Quantitative masking risk measures based on volumetric breast density measurements were computed. For this purpose, a research version of the commercial software Volpara (v1.5.0, Volpara Health Technologies, Wellington, New Zealand) was used, which provides quantitative breast density maps in addition to the percentage of dense tissue volume. In these density maps, the pixel intensity is mapped to the fibroglandular tissue thickness at each pixel location.

Three different automated measurements were investigated to estimate masking risk: (1) percent dense volume (PDV), defined as the fibroglandular tissue volume divided by the breast volume; (2) percentage dense area (PDA), computed as the percentage area on the density map where the dense tissue thickness exceeded 1 cm; and (3) a dense tissue masking model (DTMM) in which the size distribution and cancer location probability are taken into account. The idea behind the second method is that a certain amount of fibroglandular tissue is necessary to hide a cancer. With the threshold of 1 cm, the size of a region where cancers may be masked is estimated and we assume that the relative area of this region is related to masking risk. A strength of this method is the simplicity. In the third method, this idea is refined with the tissue masking model which captures two aspects. First, instead of using a fixed thickness threshold, it is modeled that larger cancers require more dense tissue to be masked than smaller cancers. For this, the normalized distribution of breast cancer size is taken into account. Second, the probability distribution of cancer location is used to take into account that dense tissue presence in regions where cancers more often occur should give a stronger increase in masking risk than dense tissue presence elsewhere. A detailed description is in the section “Appendix”.

The methods were applied to all available images in an exam, i.e., MLO and CC views of both breasts. If CC views were missing, their results for the different methods were imputed. This was done for each method separately using linear regression analysis in controls with both MLO and CC views available. To come to a single score per exam, results were averaged over the four views.

Next to the automated measurements, for the purpose of this study, the breast density category of every exam was assessed by a radiologist (10 years of experience in breast imaging) according to the fifth edition of the BI-RADS atlas [21]. Mammograms were evaluated without knowledge of the cancer status.

To implement supplemental screening strategies in clinical or screening practice, it is necessary to divide the women into two groups: women at low masking risk and women at high masking risk. In practice, a threshold needs to be determined and all women with a measure above the threshold would receive additional imaging. The best threshold is unknown for the automated measures and depends on the screening population and the proportion of women that one is willing to offer supplemental screening or the number of interval cancers that should be detected with additional imaging.

To measure to what extent the methods can identify women at high masking risk, the mammograms were divided in a high and low masking risk group by thresholding the risk measure. Then, the sensitivity of the masking measures was computed as the number of interval cancers in the high-risk group divided by the total number of interval cancers. The false positive rate is calculated as the percentage of normal controls selected as at high masking risk at the same threshold. In the context of risk stratification for supplemental screening, the proportion of controls selected as at high masking risk can be seen as supplemental screening rate and the proportion of interval cancers gives an estimate about the cancers that might be detectable with additional imaging at that supplemental screening rate.

The automated masking measures were compared to the radiologist scores when distinguishing BI-RADS density a and b versus BI-RADS density c and d. Bootstrapping was used to obtain 95% confidence intervals (CIs) and derive *p- *values.

Since breast density is a risk factor for breast cancer [14–16], it may be expected that the average breast density of women with cancer is higher than that in controls. Consequently, any predictive value of PDV for the presence of interval cancers might be caused by PDV being a risk factor, rather than being a ‘masking factor’. To investigate the potential impact of this effect on our results, an additional experiment was conducted in which it was tested to what extent PDV can distinguish women with any breast cancer from controls. Again, cases with the highest PDV were selected by thresholding, and the proportion of cancers as a function of the proportion of controls selected was computed. For this experiment, mammograms of the screen-detected breast cancers and the interval cancers detected within 24 months were used. Only the interval cancers detected later than 24 months after the last examination were not used, as we assume that these cancers might well have been detected when women would have attended another screening round.

## Results

The mean age of interval cancers and controls is 57.7 and 59.2 years, respectively. In 14.4% of the interval cancers, the cancer was diagnosed after first participation in the screening program, while 15.2% of the controls belong to women who attended the screening program for the first time. Only 3 interval cancers (2.7%) were diagnosed in women older than 70 years, while 110 women (11%) of the control group were older than 70 years.

**Fig. 1:**
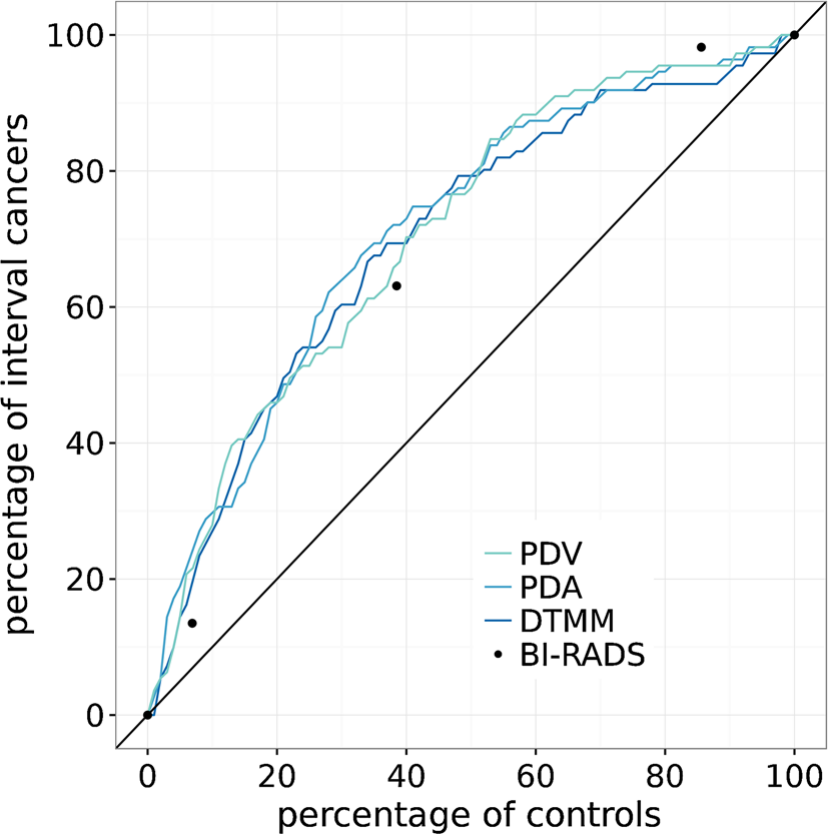
By thresholding the masking measures, cancers and controls were separated into high- and low-risk groups. The percentages of cancers and controls in the high-risk group are plotted against each other as function of the threshold

In Fig. 1, the percentage of interval cancers selected as at high masking risk is plotted against the percentage of controls selected when thresholding the different masking measures. As mentioned earlier, the proportion of controls selected as at high masking risk can be interpreted as the supplemental screening rate when a masking measure would be used in practice to identify women eligible for supplemental screening. The percentage of interval cancer selected as at high masking risk is a measure for the potential benefit of supplemental screening, since it is the proportion of women with interval cancers that would have been included in the selection if supplemental screening had been offered. The percentage of interval cancers selected for several supplemental screening rates is given in Table 1, while the supplemental screening rate required to include a certain percentage of women with interval cancer is presented in Table 2.

**Table 1:** On the masking measures, a threshold can be applied to separate cases and controls into a high- and a low-risk group. By adjusting the threshold on a masking measure, the percentage of controls (also interpretable as supplemental screening rate) is adjusted. The percentage of interval cancers that would be included in the selection at several supplemental screening rates is given in this table. Using BI-RADS breast density c and d as high-risk categories, 38.5% of the controls are considered at increased masking risk and 63.0% of the women with interval cancer would be included in the selection. In total, 111 cancers and 1110 controls were used

Percentage of controls (‘supplemental screening rate’)	Percentage of interval cancers that would have been identified to be at high risk of masking		
	PDV	PDA	DTMM
5	14.4	18.9	14.4
10	27.9	29.7	27.0
15	40.5	34.2	40.5
20	45.9	45.9	47.7
30	54.1	64.0	60.4
38.5	66.1	71.9	69.2
40	70.3	73.0	69.4
50	77.5	79.3	79.3

*PDV* percent dense volume, *PDA* percent dense area, *DTMM* dense tissue masking model

**Table 2:** On the masking measures, a threshold can be applied to separate cases and controls into a high- and a low-risk group. The threshold on the masking measure can be adjusted such that a specific percentage of the women with interval cancer is included in the high-risk group. The corresponding percentage of controls (supplemental screening rate) is given here for several percentages of interval cancers and the different masking measures. In total, 111 cancers and 1110 controls were used

Percentage of interval cancers that would have been identified to be at high risk of masking	Percentage of controls (‘supplemental screening rate’ that should be aimed for)		
	PDV	PDA	DTMM
5	1.4	1.4	2.0
10	4.3	2.4	4.1
15	5.0	3.3	5.5
20	5.9	5.1	7.1
30	10.3	11.0	11.7
40	13.7	17.4	15.0
50	22.9	22.8	21.9
60	33.2	27.1	29.1
70	39.6	36.2	40.1
80	51.4	50.5	51.5
90	61.7	67.6	67.8

*PDV* percent dense volume, *PDA* percent dense area, *DTMM* dense tissue masking model

The density scores determined visually by the radiologist were used to differentiate non-dense breasts from dense breasts, using the BI-RADS b–c transition as threshold. With BI-RADS, 38.5% (CI 35.7–41.3) of the controls were considered dense and thus at high masking risk. Of the interval cancers, 63.0% (CI 53.5–72.0) were classified as dense, using BI-RADS. If the thresholds of the three masking measurement methods were set such that there too the proportion of controls classified as at high masking risk was 38.5%, then 66.1% (CI 55.8–76.2), 71.9% (CI 63.1–80.2), and 69.2% (60.0–77.9) of the women with an interval cancer were considered at high masking risk with PDV, PDA, and DTMM, respectively. Significantly more women with interval cancers would be included in the selection process with PDA compared to BI-RADS (*p*-value 0.022). Differences in proportions between BI-RADS and PDV, and BI-RADS and DTMM were not statistically significant with *p*-values of 0.187 and 0.067, respectively.

**Fig. 2:**
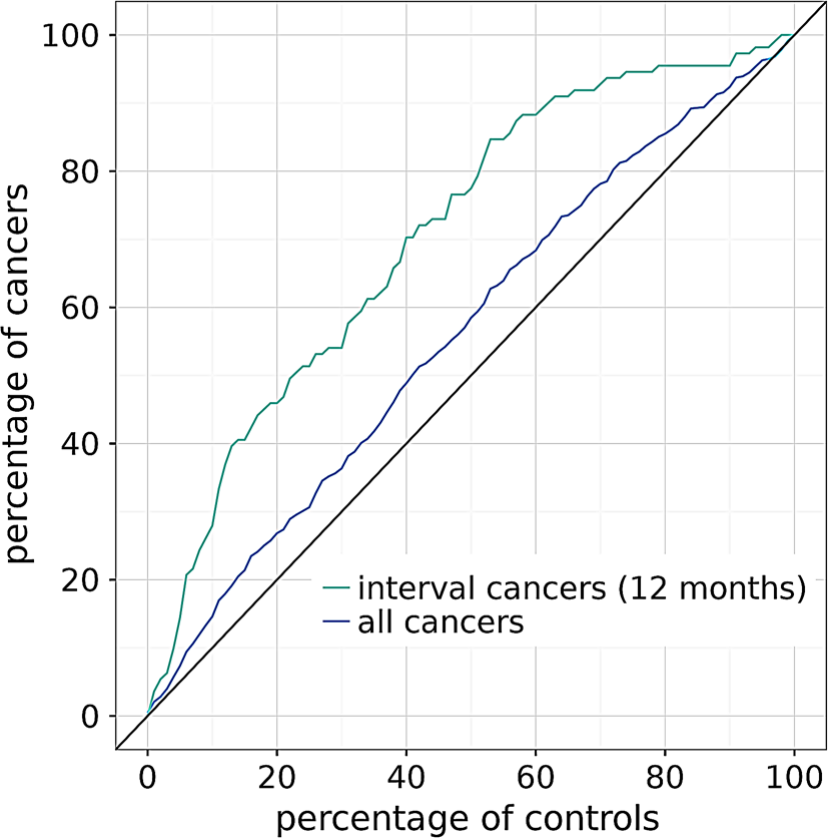
By thresholding the PDV measure, the cancers and controls were separated into a high- and a low-risk group. The figure shows the proportion of cancers and controls in the high-risk group as function of the PDV threshold

The ability of PDV to distinguish breast cancers from controls is displayed in Fig. 2. The cancers detected at a screening examination (*N* = 836) and the interval cancers that were diagnosed within the screening interval of 24 months after a negative screening examination (*N* = 275) were eligible for the analysis (*N* = 1111). The PDV estimate was available for 1103 cancers. The curve for predicting interval cancers shows a much higher area under the curve than the curve predicting all breast cancers. These results show that PDV is not ‘just’ a predictor for breast cancer risk, but in particular a good predictor for the risk of developing an interval cancer (as a proxy for risk of masking).

## Discussion

In this study, we investigated the ability of several measurements of masking risk to distinguish false negative screening mammograms from true negative screening mammograms. The aim of our work is to find a method that is suited to identify women who are at high risk to be diagnosed with an interval cancer after a negative screening exam. In a personalized screening workflow, such a method could be applied to all negative screening mammograms to select the subgroup of women who would benefit most from additional imaging with MRI or ultrasound.

There are various reasons why interval cancers are not detected by screening, and masking is only one of them. Some cancers may be not detected by screening because they grow fast and the screening interval is too long. As we focus in this study on masking, we included in our experiment only those interval cancers that were diagnosed within 12 months after the negative mammogram, to exclude true interval cancers, the cancers that show no signs of abnormality on the mammogram. True interval cancers are more common in the second year after the examination than in the first year [26]. Given that the exact cancer location was unknown and that the diagnostic mammograms were not available, it was not possible to review the interval cancers and to confirm that masking is the cause for a cancer diagnosis outside the screening program. It is noted that by excluding the interval cancers after 12 months, our study results are also more representative for screening programs with a 1-year interval.

In current clinical practice, the BI-RADS density assessment categories are used to decide whom to offer supplemental screening. Using a separation into a low-risk group (BI-RADS density a and b) and high-risk group (BI-RADS density c and d) with a 38.5% supplemental screening rate, it was found that between 63.0 and 71.9% of the women diagnosed with an interval cancer in the study data within 12 months of a negative screening would be included in the high-risk group for additional imaging. Automated measures have a higher sensitivity than the radiologist, and this difference was statistically significant for the new proposed measurements PDA at the chosen supplemental screening rate.

We compared the ability of PDV to distinguish cancers (screen-detected and interval) from controls to make sure that we capture more than the breast cancer risk in relation to breast density. Thereby, we confirmed that cancers are more common in dense breasts than in non-dense breasts. Nevertheless, we can conclude that the differences in PDV distributions of interval cancers and controls are not only caused by the increased breast cancer risk that is associated with an increased breast density, and that PDV is capturing masking.

Cancers and controls were only matched for the month of acquisition and not for age and participation in the breast cancer screening program. The mean age of the controls was higher than the mean of the cases. Given that breast density decreases with age [27], one could argue that the difference in density distribution between cases and controls is caused by differences in age. However, the control group contained more women who participated in the screening program for the first time than the cases, leading to an effect in the opposite direction. While only three interval cancers (2.7%) were found in women between 71 and 75 years of age, 11% of the controls belong to this age group causing the higher mean age in controls. If we had matched for age at the time of acquisition, women above 70 years would have been underrepresented in the controls, and the controls would have been not representative for the screening population.

Mainprize et al. [28] have been working on the quantification of masking as well. In their model, a detectability map is created for each pixel location by simulating lesions and by using local estimates of the noise power spectrum and volumetric breast density. They validated the masking measurement with an observer study on regions of interest of 150 cancer free CC mammograms. High correlations were found between the mean value of the detectability maps and the computerized and human observer study. However, Mainprize only used cancer free mammograms in his study and simulations in regions of interests. Hence, it remains an open question to which extent the mean value of the detectability map differs between false and true negative screening mammograms and whether it can be used as a predictive masking score.

A limitation of our study is the fact that CC images were not available for all exams. Until recently, MLO was the standard view in the screening program where we acquired our data, while CC images were obtained by indication. Therefore, to avoid bias when averaging over views, we imputed data for missing CC views based on the available MLO view and statistical analysis of differences between MLO and CC views. Furthermore, cases and controls were matched to the month of acquisition to guarantee the same guidelines and circumstance in image acquisition with regard to taking the CC views. Another limitation is that BI-RADS density assessments of only one radiologist were available. Many studies found inter- and intra-reader variability in breast density assessment using BI-RADS [29–32]. Therefore, to make a definitive comparison between the automated methods and radiologists assessments, an extensive reader study should be conducted with multiple readers.

In conclusion, results suggest that the new proposed masking risk measurements may have a better performance than visual BI-RADS assessment in distinguishing false negative screening mammograms from true negative screening mammograms. Therefore, these measurements may be considered as predictive masking measure when implementing supplemental screening for women at a high risk for interval cancers.

## Appendix

The dense tissue masking model (DTMM) captures two aspects: (1) the larger the lesion, the lower the masking risk; and (2) the larger the dense tissue thickness at a location, the higher the masking risk. Therefore, the masking risk for a lesion with diameter *d*_t_ at a location with density *d* is defined as

$${\text{masking}}\,\left( {d_{\text{t}} ,d} \right) = \frac{{d - d_{\text{t}} }}{d}\quad{\text{if }}\quad d \ge d_{\text{t}}.$$

From this, the masking risk for each pixel location (*x*,*y*) is estimated by summing over all possible tumor diameters considering the normalized cancer size distribution *s*(*d*_t_).

The size distribution was obtained for invasive masses. In the mammograms, a contour was drawn around the mass and the effective diameter was determined (diameter = 2 (area/π)^1/2^). The distribution was normalized to a value of one. With the use of the size distribution, we take into account that lesions with the size of few millimeters are in general not detectable and that extremely large cancers are not common in screening.

The masking risk at a location(*x*,*y*) with density *d*(*x*,*y*) is then

$${\text{masking}}\, \left( {x,y} \right) = \mathop \sum \limits_{{d_{\text{t}} = 0}}^{{d_{\text{t}} = d(x,y)}} s\,(d_{\text{t}} ) \times {\text{masking}}\, (d_{\text{t}} ,d(x,y) )$$

$${\text{masking}}\, (x,y )= \mathop \sum \limits_{{d_{\text{t}} = 0}}^{{d_{\text{t}} = d(x,y)}} s\,(d_{\text{t}} ) \times \frac{{d(x,y) - d_{\text{t}} }}{d(x,y)}.$$

Furthermore, the cancer location probability distribution (CLPD) is taken into account. With the use of the CLPD, it is acknowledged that lesions are more common in the center of the breast and less common close to the periphery. The CLPD was as well obtained with the invasive mass-like lesions as described in [33]. The probability of masking is then

$${\text{masking}}\, (x,y )= {\text{CLPD (}}x,y )\times \mathop \sum \limits_{{d_{\text{t}} = 0}}^{{d_{\text{t}} = d(x,y)}} s\,(d_{\text{t}} ) \times \frac{{d(x,y) - d_{\text{t}} }}{d(x,y)}.$$

With the formula above, the masking risk is determined for each pixel location (*x*,*y*). To come to a single score for each image, the masking risk is averaged over all pixels within the breast. Different CLPDs and size distributions were used for the MLO and CC images.
